# Care-seeking patterns for fatal non-communicable diseases among women of reproductive age in rural northwest Bangladesh

**DOI:** 10.1186/1472-6874-12-23

**Published:** 2012-08-15

**Authors:** Shegufta S Sikder, Alain B Labrique, Barkat Ullah, Sucheta Mehra, Mahbubur Rashid, Hasmot Ali, Nusrat Jahan, Abu A Shamim, Keith P West, Parul Christian

**Affiliations:** 1 Department of International Health, Johns Hopkins Bloomberg School of Public Health, Baltimore, MD, USA; 2 Maternal and Newborn Health Program, United Nations Fund for Population Activities, Narail, Bangladesh; 3 Partners in Population and Development, Dhaka, Bangladesh; 4 The JiVitA Maternal and Child Health Research Project, Gaibandha, Bangladesh; 5 Eminence Bangladesh, Dhaka, Bangladesh

## Abstract

**Background:**

Though non-communicable diseases contribute to an increasing share of the disease burden in South Asia, health systems in most rural communities are ill-equipped to deal with chronic illness. This analysis seeks to describe care-seeking behavior among women of reproductive age who died from fatal non-communicable diseases as recorded in northwest rural Bangladesh between 2001 and 2007.

**Methods:**

This analysis utilized data from a large population-based cohort trial in northwest rural Bangladesh. To conduct verbal autopsies of women who died while under study surveillance, physicians interviewed family members to elicit the biomedical symptoms that the women experienced as well as a narrative of the events leading to deaths. We performed qualitative textual analysis of verbal autopsy narratives for 250 women of reproductive age who died from non-communicable diseases between 2001 and 2007.

**Results:**

The majority of women (94%) sought at least one provider for their illnesses. Approximately 71% of women first visited non-certified providers such as village doctors and traditional healers, while 23% first sought care from medically certified providers. After the first point of care, women appeared to switch to medically certified practitioners when treatment from non-certified providers failed to resolve their illness.

**Conclusions:**

This study suggests that treatment seeking patterns for non-communicable diseases are affected by many of the sociocultural factors that influence care seeking for pregnancy-related illnesses. Families in northwest rural Bangladesh typically delayed seeking treatment from medically certified providers for NCDs due to the cost of services, distance to facilities, established relationships with non-certified providers, and lack of recognition of the severity of illnesses. Most women did not realize initially that they were suffering from a chronic illness. Since women typically reached medically certified providers in advanced stages of disease, they were usually told that treatment was not possible or were referred to higher-level facilities that they could not afford to visit. Women suffering from non-communicable disease in these rural communities need feasible and practical treatment options. Further research and investment in adequate, appropriate care seeking and referral is needed for women of reproductive age suffering from fatal non-communicable diseases in resource-poor settings.

## Background

Over the past few years, NCDs in South Asia have received increasing attention. In 2007, non-communicable diseases (NCDs) were reported to represent the highest cause-specific mortality burden among adults in Bangladesh [1]. A study conducted in two rural and two urban areas of Bangladesh between 1987 and 2002 by Molla and colleagues suggested that the prevalence of morbidity due to communicable diseases declined from 58 to 35%, while NCD morbidity increased from 33 to 57% [2]. Studies across South Asia suggest that prevalence of NCDs such as coronary heart disease and diabetes are increasing in both rural and urban populations among women and men [3,4]. Understanding the factors contributing to non-communicable disease mortality is important to further reduce adult female mortality.

Although data suggest that NCDs may also represent significant mortality burden among adult females [5], few studies focus on NCDs among women of reproductive age. Moreover, data on care-seeking patterns for NCDs and sociocultural factors that affect these patterns are lacking in rural Bangladesh. Though care-seeking behaviors for maternal complications have been well studied, these behaviors among women with NCDs are not well understood.

The maternal health literature shows that women seek care for maternal complications from a variety of medically certified providers such as doctors, nurses, and midwives as well as non-certified providers, including allopathic providers, religious healers, and homeopathic and herbal providers [6-8]. High cost of treatments, inequitable access to care, and unequal power relationships between rural residents and medical providers have been cited as reasons contributing to initial care seeking from non-certified rather than certified providers [9,10]. Whether these factors, known to be important during the vulnerable period of pregnancy and childbirth, also affect care seeking for non-communicable diseases is unknown. In Pakistan, Shaikh and colleagues demonstrate that families living in the rural north often seek spiritual and faith healers for a range of adult and childhood diseases [11]. Although understanding the care-seeking patterns for NCDs is an important initial step in improving treatment options for women suffering from NCDs, few care-seeking studies have focused specifically on NCDs in rural South Asia. In this analysis, we aim to highlight the care-seeking patterns and sociocultural factors that affect care seeking for fatal NCDs among women of reproductive age in rural northwest Bangladesh.

## Methods

This study utilized data from the JiVitA-1 trial, a large randomized controlled trial conducted between 2001 and 2007 in a population of approximately 125,000 married women of reproductive age (aged 14 to 45 years) living in northwest rural Bangladesh [12]. Covering an area of approximately 435 square kilometers, the JiVitA study area is one of the largest population research sites in South Asia and is typical of rural, agrarian South Asian communities with respect to levels of infrastructure, maternal and child nutrition, and socioeconomic and health status [12,13]. The JiVitA-1 trial assessed the efficacy of antenatal-to-postnatal weekly supplementation of women with vitamin A or beta-carotene on mortality related to pregnancy, fetal loss and infant mortality. The trial, conducted between 2001 to 2007, followed a cohort of ~125,000 married women of reproductive age (aged 14 to 45 years) living in Gaibandha, Bangladesh. The details of this trial are published elsewhere [12,14]**.**

At the beginning of the JiVitA-1 trial, a population census was conducted to identify married women of reproductive age within the boundaries of the study area. Since the original trial involved pregnant women, only women of reproductive age (14 to 45 years) were considered. Non-married women were excluded due to the social taboos surrounding unwed mothers in this population [13]. Those who were pregnant or amenstrual from breastfeeding were placed on a wait list until they were eligible to become pregnant again. Newly married women were added to the registry throughout the trial. Since the goal of the parent trial was to measure the effect of micronutrient supplementation on pregnancy-related mortality, all women who were eligible to become pregnant and who gave consent for surveillance were visited every five weeks to ascertain new pregnancies. Women who became pregnant during this surveillance period were asked for consent to participate in the parent trial. Once enrolled in the parent trial, interviewers collected detailed information on woman’s background, socioeconomic status, and morbidities experienced. If a pregnant woman reported experiencing any of twenty-five severe illnesses during interviews at early pregnancy, late pregnancy, or three months postpartum, she was referred to the provider of her choice. Women who never became pregnant during the surveillance period were not approached for consent to participate in the larger trial or to proceed with detailed interviews.

Deaths among all enrolled women, regardless of pregnancy status, were recorded in a database. For all women who died while under pregnancy surveillance, physician interviewers attempted to conduct verbal autopsies. Allowing for a four-week mourning period, research physicians contacted the families of deceased women for consent to conduct verbal autopsy interviews. The verbal autopsy tools were locally adapted and pretested based on standard instruments developed by the World Health Organization (WHO). The questionnaire included sections designed to capture detailed symptoms that the woman experienced as well as an open-ended narrative allowing respondents to describe the events leading up to the woman’s death, including health providers that were sought for care. Physician interviewers were trained in rapport building, standardized procedures, and probing techniques. Interviews typically took between 45 and 90 minutes to complete [12]. The respondents consisted primarily of husbands (40%), followed by in-laws (36%), parents or siblings (15%), and neighbors or other relatives (9%).

Physician reviewers, separate from the research physicians who conducted the interviews, reviewed the verbal autopsies to assign a proximal cause of death and up to five underlying causes of death based on the signs and symptoms described in the interview. Pregnancy-related deaths (defined as deaths among women while pregnant or within 42 days of pregnancy termination, irrespective of the cause of death [15]) were excluded. This analysis focuses on non-pregnant women to understand care-seeking patterns for non-communicable diseases. Out of the 659 women who died from non-pregnancy-related causes between 2001 and 2007, physician interviewers completed 599 verbal autopsy interviews. At the time of analysis, physician reviewers had assigned causes of death for 341 of the completed verbal autopsies. From these 341 verbal autopsies, we included the 250 deaths that were determined to result from non-communicable disease.

We performed qualitative textual analysis of these 250 NCD verbal autopsy narratives using Atlas.ti version 6.1.3 [16]. First, each narrative was read by the data analyst to determine care-seeking patterns. In order to analyze factors that influenced these care-seeking patterns, a census of the narratives was conducted to document the length of each narrative, the topics covered, and the quality of information on each topic of interest (providers and places sought for care, referrals, and obstacles to receiving care). All narratives provided information on the type and number of health providers sought and were used to describe care-seeking patterns. The 203 narratives that provided data on sociocultural factors that affected care seeking were used to develop the conceptual framework. Keywords from these narratives were then coded line by line using the grounded theory approach to identify key factors. Common themes were merged (for example, “she fell ill” and “she became sick” were coded into the same category of “falling ill”). Axial coding was then applied to organize the keywords into related categories. Finally, selective coding was performed to build a conceptual framework of the sociocultural factors and delays contributing to the observed deaths. The other, less-informative narratives (n = 47) were then analyzed for contradictions or limitations to the developed conceptual model.

For this analysis, medically certified providers were defined as providers who are recognized by legal authorities, such as board-certified (MBBS) doctors, nurses, midwives, paramedics, community skilled birth attendants, or government-trained providers. Non-certified providers were defined as providers without medical certification such as village doctors, *dais* (traditional birth attendants), shamans (traditional healers), and homeopathic treatment providers [17]. This study was approved by the Johns Hopkins School of Public Health Institutional Review Board and the Bangladesh Medical Research Council. This study was nested within the JiVitA-1 trial (Clintrials.gov number NCT00198822).

## Results

Between 2001 and 2007, 659 women died from causes unrelated to pregnancy while under JiVitA-1 surveillance. In terms of parity and age at death, the 341 women whose causes of death had been assigned at the time of analysis were similar to the 318 women whose causes of death had not yet been assigned. From the 341 women with assigned cause of death, 250 deaths were determined to result from NCDs.

Among the 250 analyzed NCD deaths, cardio-vascular disease was listed as the proximal cause of death for 40% (n = 99) of overall deaths, while cancer accounted for 30% (n = 75) of overall deaths (Table 1). Hepatic failure was listed as the proximal cause of death for 7% (n = 18) of overall deaths, followed by diabetes (6%), anaemia (5%), renal failure (3%), tuberculosis (3%), gastric ulcer (2%), hepatitis (1%), malnutrition (1%), epilepsy (1%), and lupus (1%) (Table 1). The mean age at death for women of reproductive age dying from NCDs was 35 years (standard deviation of 8 years).

**Table 1:** Causes of deaths from non-communicable diseases among 250 analyzed deaths in women of reproductive age

**Cause of Death**	**Number**	**Percentage (out of 250)**
Cardio-vascular disease	99	39.6%
* Cerebro-vascular accident*	*55*	22.0%
* Heart attack or heart failure*	*44*	17.6%
Cancer	75	30.0%
Hepatic Failure	18	7.2%
Diabetes	16	6.4%
Anaemia	13	5.2%
Renal failure	8	3.2%
Tuberculosis	7	2.8%
Gastric ulcer	6	2.4%
Hepatitis	3	1.2%
Malnutrition	3	1.2%
Epilepsy	1	0.4%
Lupus	1	0.4%

**Figure 1:**
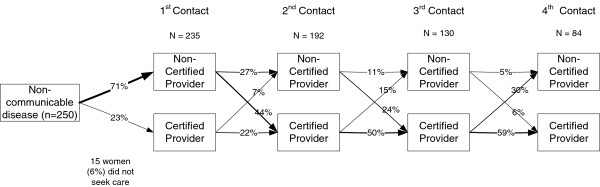
**Crossovers between certified and non-certified treatment providers among women dying of fatal non-communicable diseases (n = 250).** Figure 1 illustrates the convoluted care-seeking pathways for non-communicable diseases. Among women dying of fatal NCDs, 71% first went to non-certified providers, while approximately 23% first sought care from certified providers. Of the 192 women who sought a second stage of care, 66% sought certified medical treatment, and 34% sought treatment from non-certified providers. There were more crossovers from non-certified treatment to certified treatment rather than vice versa at each stage of treatment except for the final stages. In the final stages of care seeking, women often resorted to palliative care from homeopathic providers when they were told that no further medical treatment could be provided due to the advanced stage of disease.

From the 250 women dying of fatal NCDs in this analysis, an overwhelming majority (94%, n = 235) sought care from at least one provider. Seventy-one percent of women (n = 177) first went to non-certified providers, while 23% (n = 58) first sought care from certified providers (Figure 1). Over half of all women (52%, n = 130) went to at least three different care providers over the course of their illness. More than a third of women (34%, n = 84) went to at least four different treatment providers. Of the 192 women who sought a second stage of care, 66% sought certified medical treatment while 34% sought treatment from non-certified providers (Figure 1). Of those who initially sought medical treatment, only 7% sought treatment from non-certified providers at the second stage. There were more crossovers from non-certified treatment to certified treatment at each stage of treatment except for the final stages. In the final stages of care seeking, women often resorted to palliative care from homeopathic providers when they were told that no further medical treatment could be provided due to the advanced stage of disease.

**Figure 2:**
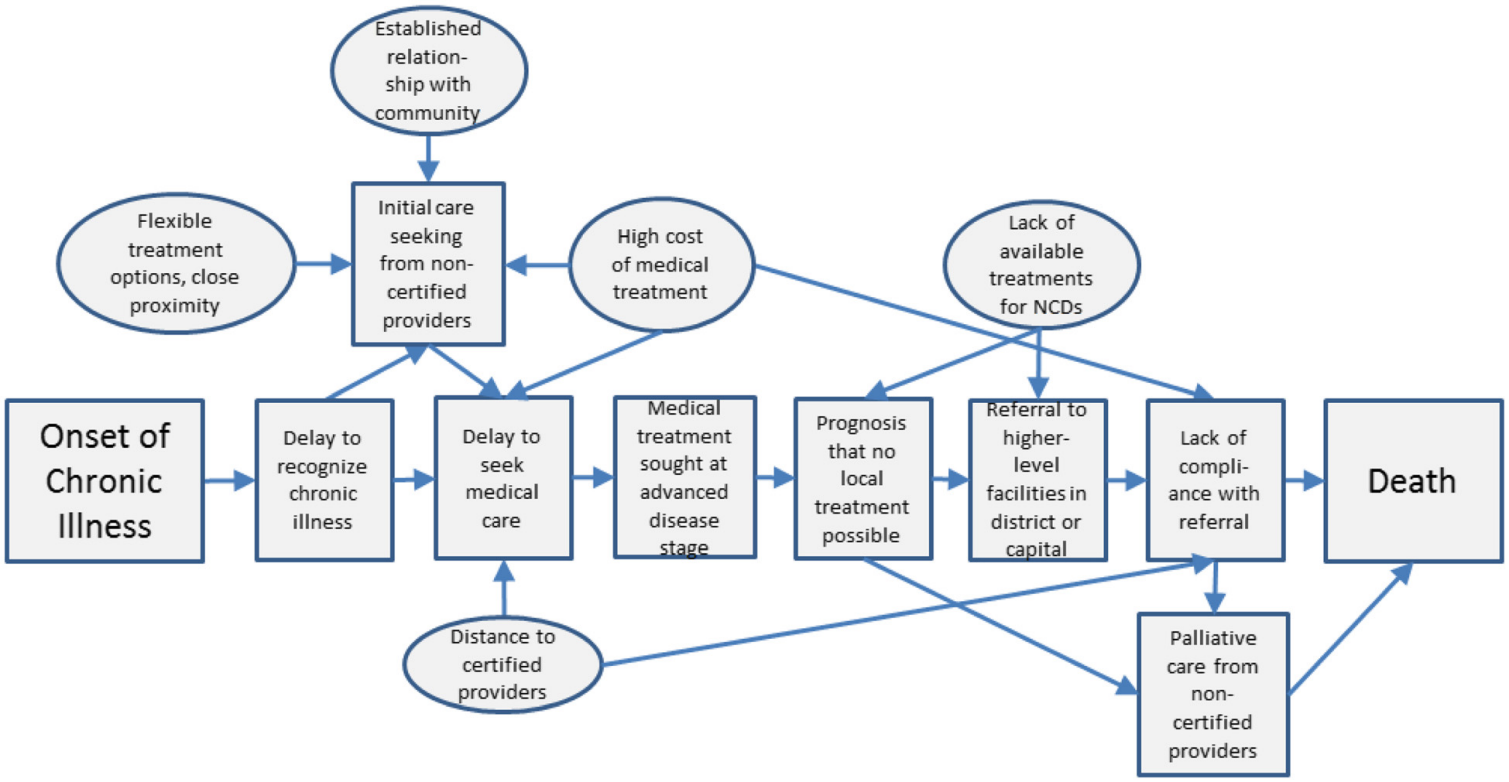
**Conceptual model of pathways to mortality for non-communicable disease deaths (n = 250).** This figure presents an overall conceptual model of common factors associated with NCD deaths. The square shapes denote actions taken by women and their families on the pathway from the onset of chronic illness to death. Circles represent factors contributing to these actions, as identified through analysis of verbal autopsy narratives. A failure to recognize the chronic nature of illness was a common theme that contributed to convoluted treatment seeking pathways. Families first sought care from non-certified providers due to flexible payment options, proximity of non-certified care providers, and established relationships with non-certified providers. Since non-certified providers usually failed to treat chronic conditions successfully, most women sought treatment from medically certified providers at advanced stages of disease. Doctors typically said that the disease had progressed to such an advanced stage that treatment could not be provided locally. Instead, they usually referred patients to higher-level facilities in district hospitals or the capital city.

In Figure 2, we present an overall conceptual model of common factors associated with NCD deaths. The square shapes denote actions taken by women and their families on the pathway from the onset of chronic illness to death, while circles represent factors contributing to these actions. A failure to recognize the chronic nature of illness was a common theme that contributed to convoluted treatment seeking pathways. Often, the families and the women themselves did not realize that they were suffering from a chronic illness. The narrative analysis revealed that families first sought care from non-certified providers due to flexible payment options, proximity of non-certified care providers, and established relationships. Local “village doctors”, the most commonly sought providers, typically visited patients at home to provide saline injections or advice on medicines. Traditional healers, the second most commonly consulted non-certified treatment providers, usually recited blessings or religious scriptures and provided amulets as treatment.

Since non-certified providers usually failed to treat chronic conditions successfully, most women sought treatment from medically certified providers at advanced stages of disease. According to respondents, doctors typically said that the disease had progressed to such an advanced stage that treatment could not be provided locally. Instead, doctors usually referred patients to higher-level facilities in district hospitals or the capital city. Many patients resorted to home-based palliative care in these later stages of non-communicable disease.

The following case illustrates a typical care-seeking pathway of women in the NCD category. This quote describes the events leading to the death of a 38-year-old woman whose proximal cause of death was determined to be cancer. “She had been sick for three months with abdominal swelling. In the third month, we saw a large round growth on her stomach. We [the woman’s family] called a village doctor at home. He came and gave her saline. When she didn’t get well, she was taken to MBBS [board-certified] doctors…. After receiving treatment for 15 days, she was sent to a clinic for an ultrasonography test. After reviewing the report, the doctor said that he would not be able to provide treatment and admitted her to a government hospital. After staying there for 12 days, she was found to have blood cancer. The doctor said that no treatment was available in the area, and that she should be taken to Dhaka [the capital city] for treatment. She was taken home due to lack of money, and she took homeopathic and herbal treatment for a few days. However, once her illness worsened, she was taken to the district medical college hospital [a hospital located approximately 48 kilometers from her home]. The doctors there told her that there was nothing they could do. She was taken home, and died two weeks later.”

The following example of a 30-year-old woman who died of renal failure is typical of a mortality pathway found in the NCD verbal autopsy narratives. The woman experienced drowsiness, fatigue, and whole body pain for five months. She initially visited a village doctor for her symptoms, but her illness did not subside. “For these symptoms, she went to the district medical college hospital, where a doctor examined her and said that she had a kidney problem. She was referred to Dhaka for better treatment. But because of poverty she was taken home. Gradually, she became sicker, with watery swelling all over her body, loss of appetite, and constant vomiting. After suffering from these symptoms for three months, she died.”

These cases exemplify the complicated care-seeking pathways in this group. Women seldom made their own health care decisions, but rather their families made all major decisions regarding the care-seeking process. Usually, families first sought care from non-certified providers to treat the woman’s illness. When these treatments failed, families typically took women to certified care providers. If these providers were unable to treat the conditions, the women were referred to higher level hospitals or private providers. For cases of cancer, doctors usually advised patients to visit the medical college hospital (located 48 km away from the center of the study area) or the capital city (approximately 270 km away) for treatment. The majority of patients were unable to comply with referral advice due to distance and cost of these options. Doctors typically said that the families had waited too long to seek care and that nothing else could be done for the woman’s health.

Seventy percent of respondents for women who died from non-communicable diseases reported poverty as a reason for not seeking medical treatment. The following narrative illustrates these barriers. In this narrative, a 30-year-old woman began having fever, swelling in her legs, and sores and nodules on her back. She received saline injections from a local village doctor, “but she was not cured. She was then admitted to a government hospital, where the doctors said that her sores were not healing because she had diabetes. They [the doctors] wrote prescriptions for medicines, but she could not buy medicine due to lack of money and was taken home. Although several herbal treatments were tried, her sores were not cured. Fluid dripped from her sores continuously. In her last months, she could not move around by herself…” Suffering in this condition, the woman died two and a half months later from what the physician reviewers determined to be diabetes. Respondents typically listed poverty as a reason for lack of compliance with prescribed treatment. If health care providers were unable to provide effective treatment at their facilities, they often referred patients to other clinics that were unaffordable for the woman and her family.

## Discussion

This study is one of the first to describe factors affecting care-seeking patterns exclusively for NCDs in rural South Asia. Medical pluralism, the practice of seeking care from both certified and non-certified providers, was reported among women dying from NCDs. This study suggests that many of the factors that affect care seeking for pregnancy-related complications, such as established relationships with non-certified providers and flexible payment schemes, also influence care seeking for NCDs. Similarly, our study suggests that women also seek multiple providers for NCDs and that they often seek certified providers in late stages of illness.

Our study confirms findings from other studies that the decision to seek care from certified providers is often delayed mainly due to financial concerns [18,19]. The data suggest that the prohibitive cost of prescribed treatments prevents women from receiving and obtaining treatment even if they visit certified providers. Health care providers often referred patients to other clinics that were unaffordable for the patient’s family. For NCDs such as cancer, physicians often referred patients to Dhaka for treatment. Yet the cost of the trip to the capital city combined with the potential loss of income during this period made this option impractical for most families. Though families often sought multiple levels of care, delays in seeking medical treatment and the inaccessibility of services for NCDs may have contributed to the lack of effective treatments provided to patients at advanced stages of disease.

This study complements findings from an analysis of care seeking for severe acute obstetric complications performed in the same study population in rural northwest Bangladesh [9]. Non-certified providers appear to be first-line providers for both NCDs and severe obstetric complications. Studies showing that non-certified providers are visited first for pregnancy-related complications, adult illnesses, childhood diseases, and also NCDs imply that non-certified providers serve as the universal initial providers for all illnesses in rural settings of South Asia [6,7,9,11].

Women suffering from NCDs may have exhibited convoluted pathways of care seeking partly due to the long time frame of disease. Program planners may be interested in decreasing the time between initial recognition of an NCD and appropriate care seeking. Further research on NCDs in rural resource-poor settings is needed to understand the factors that affect illness recognition and appropriate care seeking. Future studies may also describe the types and costs of treatments currently provided by different levels of providers.

Since this analysis is a secondary analysis of a larger intervention trial, the data collection was not specifically designed to investigate care-seeking patterns among women suffering from fatal NCDs. The narratives did not always provide complete information on the time interval between presentation of symptoms and care seeking, the types of treatment prescribed by providers, or the compliance of women with these treatments. Thus, we were unable to deduce time intervals between disease presentation and care seeking or to describe the types of treatment provided to patients. However, the narratives provided sufficient information to understand the order of providers that were sought for care.

Since non-pregnant women were not enrolled for detailed interviews on socioeconomic status or morbidities, we could not analyze care-seeking patterns by the background of the woman. As this analysis addresses fatal NCDs, the care-seeking patterns described in this paper may differ from those of women who suffer from non-fatal NCDs or those who were diagnosed early enough to prevent death. However, this analysis helps to address the lack of data on factors that affect care seeking for NCDs in rural South Asian communities. Although all verbal autopsies were not completed at the time of analysis, women whose deaths were recorded through the 341 analyzed verbal autopsies were similar to the larger group of 659 recorded deaths with respect to age at death and parity.

## Conclusions

This study provides insights into care-seeking patterns for non-communicable diseases in rural northwest Bangladesh. Women of reproductive age dying from NCDs in rural Bangladesh face similar contextual obstacles to accessing health care as women who die from maternal causes. Non-certified providers remain the first-line providers for women who suffer from NCDs due to flexible payment options, proximity, and established relationships with the community. Future studies could address data gaps on the time intervals between stages of care seeking for NCDs compared to other diseases, the types of treatment that are prescribed by various treatment providers for NCDs, and the compliance of the patients with these treatments. Data on risk factors for NCDs in women of reproductive age are needed to further illuminate the pathways to NCD mortality in this population.

Most importantly, the burden of NCD mortality among adult females deserves greater attention, especially given the suggested lack of effective strategies available to this population. Improvements in early detection, appropriate care seeking, and service delivery are necessary to advance treatment options for NCDs in resource-poor settings.

## Competing interests

The authors state that they have no competing interests.

## Authors’ contributions

SSS provided data acquisition, data analysis and interpretation, and primary drafting and editing of the article. ABL provided primary conception and design. SSS and ABL were responsible for analytic design, revision of the article, and final approval. PC, SM, MR, KPW, and AAS contributed to conception and design of the study, critical revisions of the article, and final approval. BU, HA, and NJ assisted with data acquisition, revision of the article, and final approval. All authors read and approved the final manuscript.

## Pre-publication history

The pre-publication history for this paper can be accessed here:

http://www.biomedcentral.com/1472-6874/12/23/prepub
